# Plasmalogens Rescue Neuronal Cell Death through an Activation of AKT and ERK Survival Signaling

**DOI:** 10.1371/journal.pone.0083508

**Published:** 2013-12-20

**Authors:** Md. Shamim Hossain, Masataka Ifuku, Sachiko Take, Jun Kawamura, Kiyotaka Miake, Toshihiko Katafuchi

**Affiliations:** 1 Department of Integrative Physiology, Graduate School of Medical Sciences, Kyushu University, Fukuoka, Japan; 2 Central Research Institute, Marudai Food Co. Ltd., Osaka, Japan; Winship Cancer Institute of Emory University, United States of America

## Abstract

Neuronal cells are susceptible to many stresses, which will cause the apoptosis and neurodegenerative diseases. The precise molecular mechanism behind the neuronal protection against these apoptotic stimuli is necessary for drug discovery. In the present study, we have found that plasmalogens (Pls), which are glycerophospholipids containing vinyl ether linkage at *sn-1* position, can protect the neuronal cell death upon serum deprivation. Interestingly, caspse-9, but not caspase-8 and caspase-12, was cleaved upon the serum starvation in Neuro-2A cells. Pls treatments effectively reduced the activation of caspase-9. Furthermore, cellular signaling experiments showed that Pls enhanced phosphorylation of the phosphoinositide 3-kinase (PI3K)-dependent serine/threonine-specific protein kinase AKT and extracellular-signal-regulated kinases ERK1/2. PI3K/AKT inhibitor LY294002 and MAPK/ERK kinase (MEK) inhibitor U0126 treatments study clearly indicated that Pls-mediated cell survival was dependent on the activation of these kinases. In addition, Pls also inhibited primary mouse hippocampal neuronal cell death induced by nutrient deprivation, which was associated with the inhibition of caspase-9 and caspase-3 cleavages. It was reported that Pls content decreased in the brain of the Alzheimer’s patients, which indicated that the reduction of Pls content could endanger neurons. The present findings, taken together, suggest that Pls have an anti-apoptotic action in the brain. Further studies on precise mechanisms of Pls-mediated protection against cell death may lead us to establish a novel therapeutic approach to cure neurodegenerative disorders.

## Introduction

Plasmalogens (Pls) constitute a special class of glycerophospholipids characterized by the presence of a vinyl ether linkage at the *sn-1* position of the glycerol backbone [1-4]. Pls are found in various human tissues including their major presence in the nervous, cardiovascular and immune systems [1,4]. Human heart tissues contain 30-40% choline Pls in total glycerophospholipids [5]. On the other hand, about 30% and 70% of the glycerophospholipids in human brain and in myelin sheath are ethanolamine Pls, respectively, which are much more abundant than choline Pls [5,6]. The presence of the vinyl ether bond in Pls has been suggested to play a defensive role against reactive oxygen species compared to that of ester linked glycerophospholipids [6,7]. Pls also play important roles in transporting ions through plasma membranes, membrane fusion, and the efflux of cholesterol from plasma membrane [6,7]. Furthermore, Pls are found in the cell nucleus and synaptic cleft, suggesting a wide range of functional contribution to the neuronal activity [8]. An increase in Pls levels was reported to facilitate DNA synthesis by activating phosphatidylcholine-dependent phospholipase C [8]. However, the involvement of Pls in the activation of cellular signaling to regulate growth, differentiation and apoptosis is mostly unknown.

Two peroxisomal enzymes glyceronephosphate O-acyltransferase (GNPAT) and alkyldihydroxy acetonephosphate synthase (AGPS) initiate Pls biosynthesis. Deficiency or wrong localization of these enzymes result in the severe disorder, rhizomelic chondrodysplasia punctate (RCDP), indicating that Pls are necessary for normal function of neurological, skeletal, visual, respiratory, and reproductive systems [9,10]. Reduced levels of Pls have also been associated with Alzheimer Disease (AD), X-linked adrenoleukodystrophy and Down syndrome [11-13]. These evidences give us important hints that Pls may have an important role in normal cellular function and disruption of this glycerophospholipids can lead many diseases including neurodegenerative disorders. 

In our previous study, we have reported that peripheral administration of Pls in rats can increase Pls contents in the brain and can inhibit lipopolysaccharide (LPS)-induced activation of microglia resulting in the attenuation of neuroinflammation and accumulation of β-amyloid (Aβ) protein [3]. However, the direct effects of Pls on neurons were mostly unknown. To assay the effect of Pls, we have employed apoptotic model of mouse neuroblastoma derived cell lines Neuro-2A as well as primary hippocampal neuronal culture. The experiments of the serum starvation-induced apoptosis of Neuro-2A showed that Pls had potency to inhibit cellular apoptosis. 

Our data indicated that Pls induced phosphorylation of AKT and ERK1/2 that were necessary for preventing the apoptosis. We also found that serum starvation-induced apoptosis was associated with the activation of caspase-9 but not of caspase-8 and caspase-12 and treatment with Pls blocked the activation of caspase-9 resulting in the inhibition of apoptosis. Similar evidences were also seen in primary hippocampal neuronal cultures but not mouse astrocyte-derived A1 cells. We therefore, propose for the first time that Pls has an anti-apoptotic role in the neurons. It is still unknown the molecular mechanism of Pls-mediated activation of AKT and ERK in the neuronal cells and this will be investigated in our future studies. 

## Materials and Methods

All experimental procedures involving the use of animals were approved by the Ethics Committee on Animal Experiments at Kyushu University, Japan. We have strictly followed the guidelines “Principles for the Care and Use of Animals” described by the Physiological Society of Japan. All efforts were made to minimize animal’s suffering and the number of animals used for study.

### Cell culture and Reagents

Mouse neuroblastoma derived cells, Neuro-2A (cell number: IFO50081), and astrocyte-derived cells A1 (cell number: IFO50519) were purchased from Health Science Research Resources Bank, Japan. Neuro-2A and A1 cells were maintained in MEM and DMEM medium, respectively, supplemented with 10% heat-inactivated fetal bovine serum (FBS) (Invitrogen, Carlsbad, CA, USA), 50 µg/ml penicillin, and 50 µg/ml streptomycin (Invitrogen). Cells were cultured in a humidified chamber provided with 5% CO_2_ at 37 °C. Highly pure Pls were prepared by extracting from chicken skin as reported previously [14]. Pls consisted of 96.5% ethanolamine Pls, 2.5% choline Pls, 0.5% sphingomyelin and 0.5% other phospholipids. Pls were dissolved in 99.5% ethanol to the stock concentration of 10 mg/ml and diluted to the desired concentration (5-20 µg/ml) immediately before use. Vehicle (ethanol at the same concentration without Pls) was used in the control groups. Phosphoinositide 3-kinase (PI3K)/AKT and MAPK ERK Kinase (MEK) inhibitors named LY294002 (Catalog number #9901) and U0126 (#9903), respectively, were purchased from Cell Signaling Technology (Boston, MA, USA). The dye Dil used for staining the neuronal cells and the Retinoic acid used for the apoptosis assays were purchased from Sigma. 

### DNA fragmentation assays for apoptosis

Genomic DNA was extracted from the control and experimental cells by the standard protocol. In brief, cells were washed with phosphate buffered saline (PBS). After centrifugation at 3000 rpm for 5 minutes, cell pellets were lysed with the tail lyses buffer (10 mM Tris pH 8.0, 100 mM NaCl, 10 mM EDTA pH 8, 0.5% SDS) mixed with Proteinase K and incubated at 55 °C for 12 hours. The mixture was then treated with equal volume of 5M NaCl and centrifuged to get the supernatant. Isopropanol was added to the supernatant to pull down genomic DNA. The extracted DNA was then washed by 70% ethanol solution for 2 times and dissolved with TE buffer. The genomic DNA was then separated in 0.6% agarose gel and visualized by ethidium bromide staining. 

### Fluorescence-activated cell sorting (FACS) analysis using propidium iodide (PI)

Neuro-2A cells were washed by ice cold PBS to remove floating death cells. Brief trypsinization was carried out followed by collection with PBS supplemented with 1% FBS. Propidium iodide (PI, 0.05 mg/ml) was mixed with the buffer (0.1% sodium citrate and 0.1% triton X100 dissolved in PBS. Cells were then treated with this PI buffer supplemented with RNAse for 30 minutes at room temperature in the dark followed by store in 4 °C until FACS assays. Cells were filtered with 40 μm nylon mesh before subjected into flow cytometer (BD FACS Calibur, Becton Dickinson and Company, Franklin Lakes, NJ, USA) for DNA content analysis. 

### TUNEL assays

TUNEL apoptosis assays were carried out by the provided protocol of In situ Cell Death Detection Kit, TMR red (Roche). Simply, cells were washed by PBS and fixed with 2% PFA (paraformaldehyde) for 15 minutes at room temperature. After PBS wash, cells were permeabilised by 5 minutes treatment of 0.1 % Triton X-100 in freshly prepared 0.1% sodium citrate. Cells were washed by PBS again followed by treatment of the TUNEL mixture for 1 hr at 37 °C. Residual TUNEL kits were washed properly by ice cold PBS followed by the treatment with DAPI (1 µg/ml) for 20 minutes. Cells were then mounted and analyzed by fluorescence lifetime imaging microscope (Keyence, BZ-9000 series). 

### Western blot analysis

Western blotting was performed using the whole cell lysates. Before collection, cells were washed by ice cold PBS. RIPA (radioimmunoprecipitation assay) buffer was used as lysis buffer which contained 1% NP-40, 0.5% sodium deoxycholate and 0.1% SDS dissolved in 1X TBS buffer. Protease inhibitor cocktail (Roche), and phosphatase inhibitor mixture (Sigma) was added to the RIPA buffer before cell lysis procedures. After thirty minutes on ice, cells were subjected to brief sonication on ice cold water, followed by centrifugation at 15,000 rpm for 10 minutes to remove insoluble cell debris. Protein concentration was measured by the BCA protein assay kit (Thermo Scientific) and a total of 20 to 50 µg protein were separated by 8 to 15% SDS-PAGE and electrophoretically transferred onto nitrocellulose membranes (BIO-RAD). After protein transfer, nitrocellulose membranes were blocked with Tris-buffered saline (TBS) containing 5% Difco Skim Milk (BD) and 0.1% Tween 20 for 1 hr at room temperature. Membranes were then incubated at 4 °C for overnight with the antibodies against cleaved caspase-9, caspase-9, cleaved caspase-3, cleaved caspase-12, cleaved caspase-8, caspase-8, phospho-AKT (S-473), phospho-ERK, AKT, ERK purchased from Cell Signaling Technology. Beta-actin antibody (C-4) was purchased from Santa-Cruz. Membranes were then washed after the treatments with primary antibodies and incubated with horseradish peroxidase-coupled goat anti-mouse or anti-rabbit IgG secondary antibody (Cell Signaling Technology) at room temperature for 2 h. Signals from the transferred protein onto the nitrocellulose membrane were then visualized by Super Signal West Pico Chemiluminescent Substrate (Thermo Scientific) using LAS4000 Biomolecular Imager. Quantification of the images was performed by the Image-J software.

### Cell proliferation (WST-8) assays

Number of viable cells was determined by the cell proliferation assay kit, Cell Counting Kit-8 (Dojindo, Kumamoto, Japan) following the standard protocol. The absorbance value at 450 nm was considered to be relative number of viable cells.

### Primary hippocampal neuron culture assays

Primary hippocampal neurons were prepared from E-18 embryo of mice. After dissection of anesthetized pregnant mice, meninges of embryo were removed carefully. The hippocampus were cleared with the surrounding cortex and dissolved in trypsin solution containing PBS, bovine serum albumin (BSA), and glucose at 37 °C for 15 minutes. FBS was used to neutralize the trypsin activity. The hippocampus were then dissociated in neurobasal medium (GIBCO) supplemented with B27 (GIBCO) by appropriate pipetting using different pour sized Pasteur pipette. The dissociated neurons were then cultured on poly-D-lysine coated glass cover slips (30,000 cells/15 mm coverslip) with neurobasal medium in 5% CO_2_ humidified incubator. On DIV (Disk In Vitro) 3, 90% of cultured medium was replaced with B27 free neurobasal medium with or without Pls (5 µg/ml). Cytosine arabinoside (Ara-C) purchased from Sigma was added on DIV 3 primary neurons at a concentration of 1 µM to inhibit microglial proliferation. At DIV 6, neurons were stained by the dye dil and photographed by Keyence fluorescence lifetime imaging microscope. 

### Immunocytochemistry assays

Primary hippocampal neurons of DIV6 were subjected to immunohistochemistry by the protocol described below. Cells were fixed by 4% PFA for 20 minutes at room temperature followed by PBS wash for three times (5 minutes each). Cells were then incubated by the blocking buffer containing cell permeabilizing detergent Triton-X100 for 1 hr. The blocking buffer is composed of 0.2% TritonX-100 and 5% BSA dissolved in PBS. After PBS wash for 3 times, cells were then incubated with the primary antibodies of NeuN (1:2000, Millipore), Cy3 conjugated GFAP (1:2000, Sigma), and Iba-1 (1:2000, Wako) dissolved in the blocking buffer at 4 °C for 24 hours. PBS wash was carried out followed by the treatments with the Alexa Fluor 488/568 conjugated secondary antibody (1:1000, Life Technologies) dissolved in the blocking buffer for 2 hours at room temperature. After incubation with secondary antibodies cells were washed and treated with DAPI solution (1 μg/ml in the blocking buffer) for 10 minutes at room temperature. After single washing by PBS, cells were mounted by PermaFluor aqueous mounting medium (Thermo Scientific) and analyzed by fluorescence lifetime imaging microscope (Keyence, BZ-9000 series). 

### Statistics

The data were expressed as mean ± SEM. Effects of serum starvation and drugs were analyzed by one-way analysis of variance (ANOVA) followed by Bonferroni’s post hoc test.

## Results

### Plasmalogens inhibit apoptosis of neuronal cells upon serum starvation

**Figure 1:**
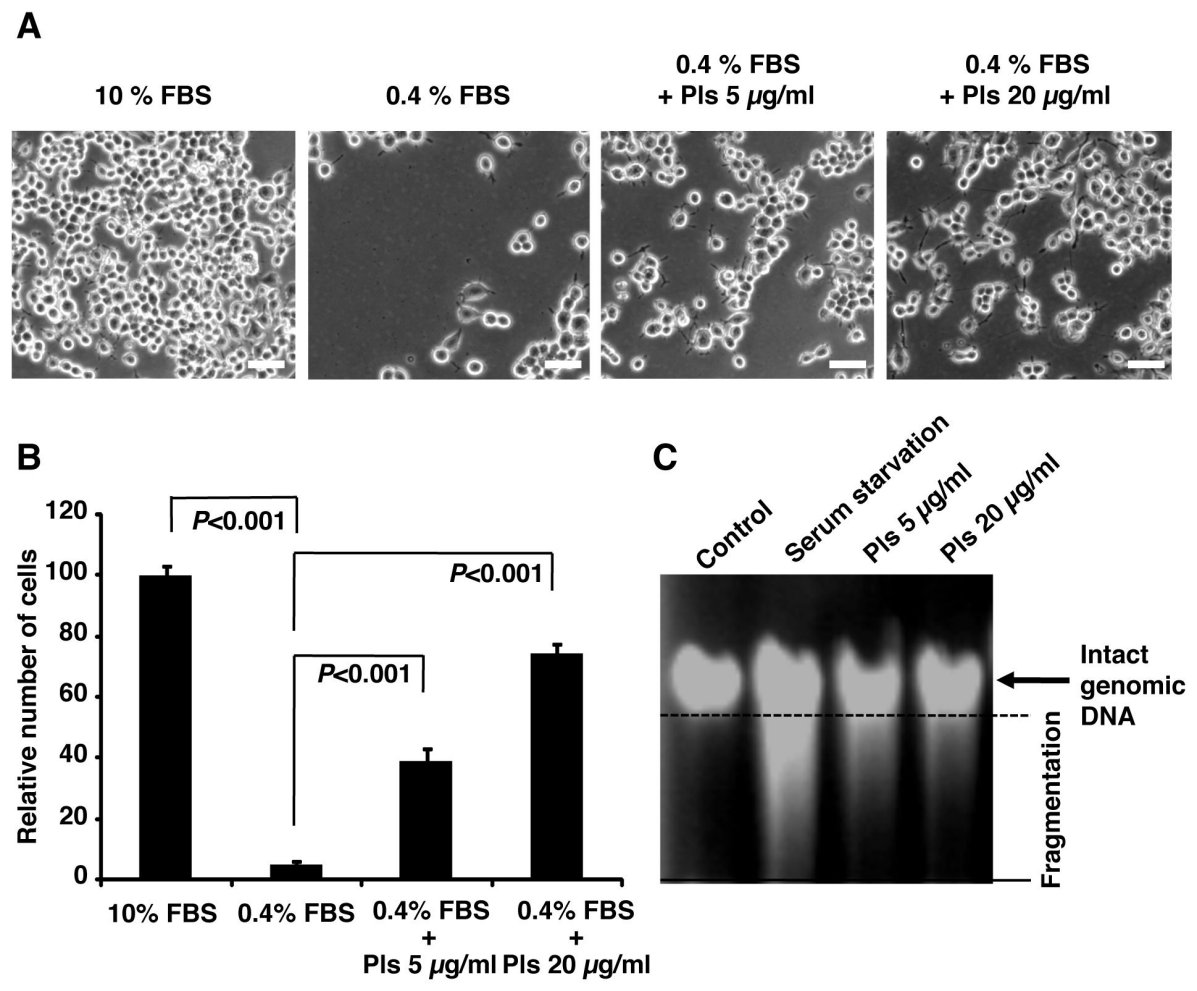
Plasmalogens inhibit apoptosis of Neuro-2A cells upon serum starvation. **A**) Neuro-2A cells were treated with 10% FBS, 0.4% FBS, 0.4% FBS plus plasmalogens (Pls) of concentrations 5 µg/ml and 20 µg/ml for 72 hours. Cells were then washed by culture medium and photographs were taken by the microscope. Scale bar, 50 µm. **B**) Quantification of the survival cells as in panel A was done by trypan-blue exclusion methods using hemocytometer. Statistical significance between groups was calculated by one-way ANOVA followed by Bonferroni’s test using SPSS software. **C**) DNA fragmentation assays were carried out 72 hours following serum starvation w or w/o the Pls treatments. Pls treatments inhibited the DNA fragmentation.

**Figure 2:**
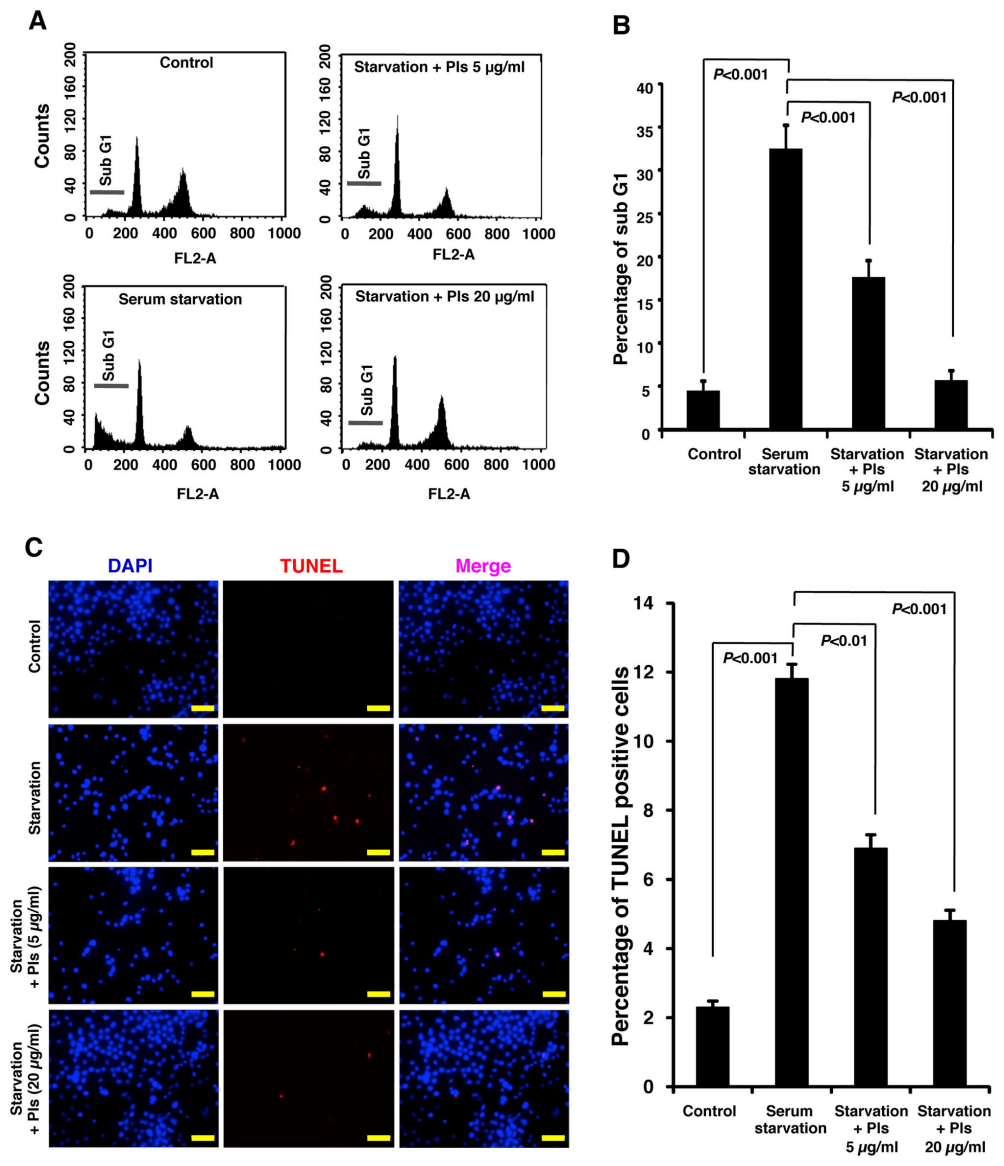
Plasmalogens reduce sub-G1 population and TUNEL positive Neuro-2A cells. **A**) Neuro-2A cells were cultured in 10% FBS (control group), 0% FBS (serum starvation) and 0% FBS plus Pls of 5 and 20 µg/ml for 36 hours. FACS assays was performed by PI staining. **B**) Quantification of the FACS histograms showed a significant reduction of sub-G1 cells population by Pls compared with the control group upon the serum starvation (n=3, *P*<0.001, Bonferroni’s test). **C**) TUNEL staining shows the reduction of TUNEL positive cells by Pls in the serum starved groups. The experimental condition was similar with that of panel A. Scale bar, 50 µm. **D**) Pls treatments significantly inhibited TUNEL positive cells (n=5, *P*<0.01, Bonferroni’s test). Histograms show the average number of TUNEL positive cells in percentage of each group.

Reduced level of Pls has been reported to be associated with AD. We therefore, hypothesized that Pls might have protective effects on neurons. To investigate the role of Pls in neuronal apoptosis, we subjected Neuro-2A cells to serum starvation. After 72 hours culturing with MEM medium supplemented with very low serum (0.4% FBS), Neuro-2A cells underwent apoptosis (Figure 1A). To quantify the apoptosis, we counted the live cells by trypan-blue exclusion methods. Following 72 hours, serum starvation significantly reduced the viable cells (Figure 1B, each group, n=4, *P*<0.001, Bonferroni’s post hoc test). Interestingly, addition of Pls (5 and 20 μg/ml) significantly rescued neuronal cell death caused by serum starvation (Figure 1B) (n=4, *P*<0.001). We have also employed Genomic DNA fragmentation assays to confirm apoptosis. In the same experimental condition, Pls treatments reduced DNA smearing caused by serum starvation (Figure 1C). The cell proliferation study, WST-8 assays, also showed that Pls at the dose of 5 and 20 µg/ml could effectively restore the viability of Neuro-2A cells (data not shown). To further examine the effect of Pls on cellular survival upon serum starvation, we have employed FACS analysis using PI. FACS data showed an increase of Sub-G1 cell population upon serum starvation and Pls administration effectively reduced these cell populations (Figure 2A). DNA content was quantified from the FACS histograms to calculate the percentage of sub-G1 cell population. As shown in Figure 2B, serum starvation significantly increased the number of Sub-G1 cell population and Pls treatments reduced the sub-G1 cell population significantly in a dose dependent manner (each group, n=4, *P*<0.001), suggesting an anti-apoptotic role of Pls in the neuronal cells.

It has been well known that the sub-G1 cell populations can also include necrotic cells along with the apoptotic cells. Therefore, to specify the anti-apoptotic role of Pls, we have further employed the popular apoptotic assays, TUNEL staining. In consistence with the DNA fragmentation and FACS assays, Pls significantly reduced the TUNEL positive cells (Figure 2C and D) (n=4, *P*<0.01 and 0.001, Bonferroni’s test). 

### Plasmalogens inhibit cleavage of the caspases

**Figure 3:**
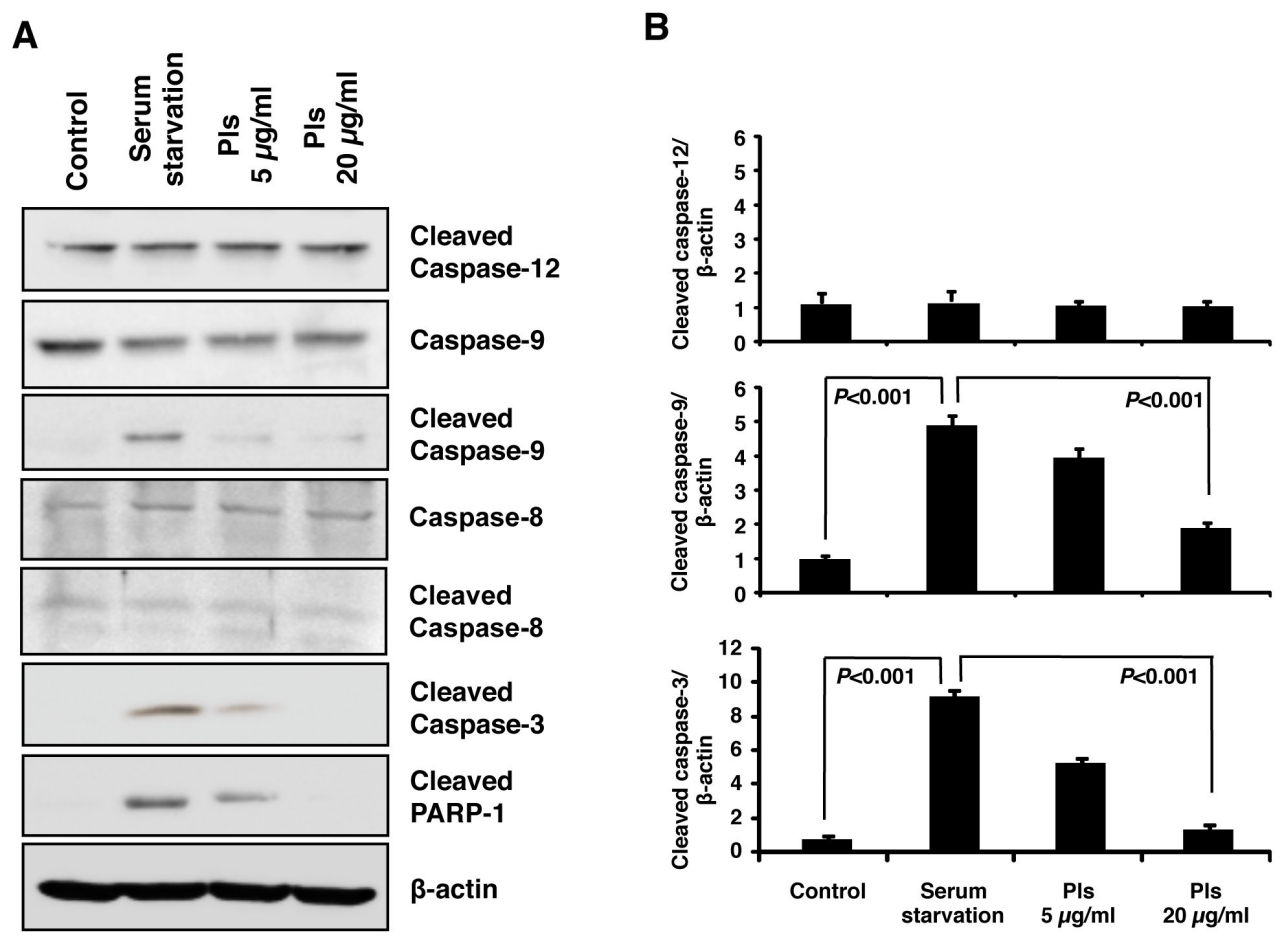
Plasmalogens inhibit cleavage of caspase-9 and caspase-3. **A**) Serum starvation increases cleavage of caspase-9 but not of caspase-8 and caspase-12. Neuro-2A cells were cultured in the culture medium supplemented with 10% FBS (control group), 0% FBS (serum starvation) and 0% FBS plus Pls of 5 and 20 µg/ml for 72 hours. Cells extracts were then subjected to western blotting with the specific antibodies against caspases. Fifty microgram proteins of each group were analyzed. **B**) Pls significantly inhibit cleavage of caspase-9 and caspase-3 (each group, n=4, *P*<0.001, Bonferroni’s test). Image-J software was used to quantify the signals.

There are several caspases that are reported to be involved in serum starvation-mediated apoptosis. To identify the caspases involved in our apoptosis model of Neuro-2A cells, we have performed western blot assays. In our experimental condition, Pls treatments at the doses of 5 and 20 µg/ml effectively inhibited the apoptosis of Neuro-2A cells following 72 hours serum starvation (Figure 1). We therefore, used the similar experimental condition to identify the possible caspases associated with the serum starvation. Western blotting assays using the specific antibodies to detect activation of intrinsic apoptotic pathway (caspase-9), extrinsic apoptotic pathway (caspase-8) and endoplasmic reticulum (ER) stress-mediated apoptotic pathway (caspase-12) were carried out. In our experimental condition, cleavages of caspase-8 and caspase-12 upon serum starvation were not changed (Figure 3A). Efficiency of the caspases antibodies was confirmed by appropriate positive control experiments to exclude the possibility of artifacts (data not shown). As shown in Figure 3A, only cleaved caspase-9, which represents mitochondria-mediated intrinsic apoptosis pathway, was increased by serum starvation. This cleavage of caspase-9 has been reported to induce cell death by activating the downstream caspase-3 and PARP-1 (poly ADP-ribose polymerase-1). In consistence with this, our serum starvation effectively induced cleavage of both caspase-3 and PARP-1 (Figure 3A). Very interestingly, Pls treatments reduced the cleaved caspase-9, caspase-3 and PARP-1 (Figure 3A). Quantification of the western blotting images showed that Pls significantly inhibited cleavages of caspase-9 and caspase-3 (Figure 3B) (each group, n=4, *P*<0.001). These evidences support that apoptosis of Neuro-2A cells induced by serum starvation is associated with the intrinsic pathway and Pls effectively block this apoptotic pathway. 

### Plasmalogens enhance phosphorylation of AKT and ERK1/2

**Figure 4:**
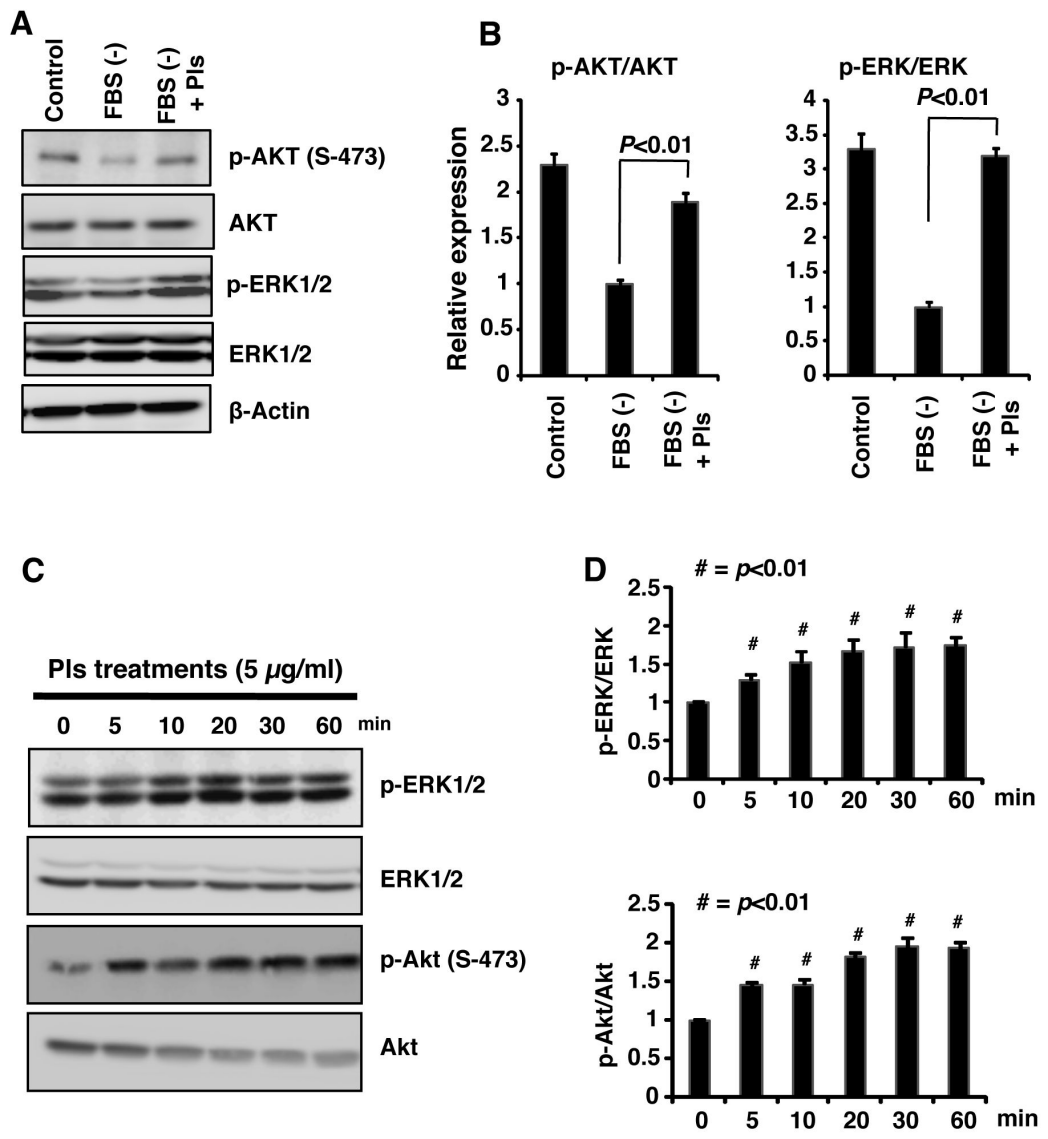
Plasmalogens enhance phosphorylation of AKT and ERK1/2. **A**) To see the signaling pathways, Neuro-2A cells were cultured in 10 % FBS (Control) and 0% FBS with or without Pls (5 µg/ml) for 24 hours. Whole cell extract (50 µg protein) was subjected to western blotting (left). All the data represents more than three independent experiments. **B**) Quantification of the data showed that Pls treatments significantly increased the phosphorylation of AKT and ERK1/2 compared with the serum free group (each group, n=4, *P*<0.01, Bonferroni’s test). **C**) Pls significantly increase phosphorylation of Akt and ERK in the increasing time points (each group, n=4, *P*<0.01, Bonferroni’s test). Neuro-2A cells were cultured in serum free medium for 12 hours followed by treatments with Pls (5 µg/ml) for the indicated time periods. Western blotting data showed a time dependent increase in the phosphorylation of AKT and ERK1/2.

**Figure 5:**
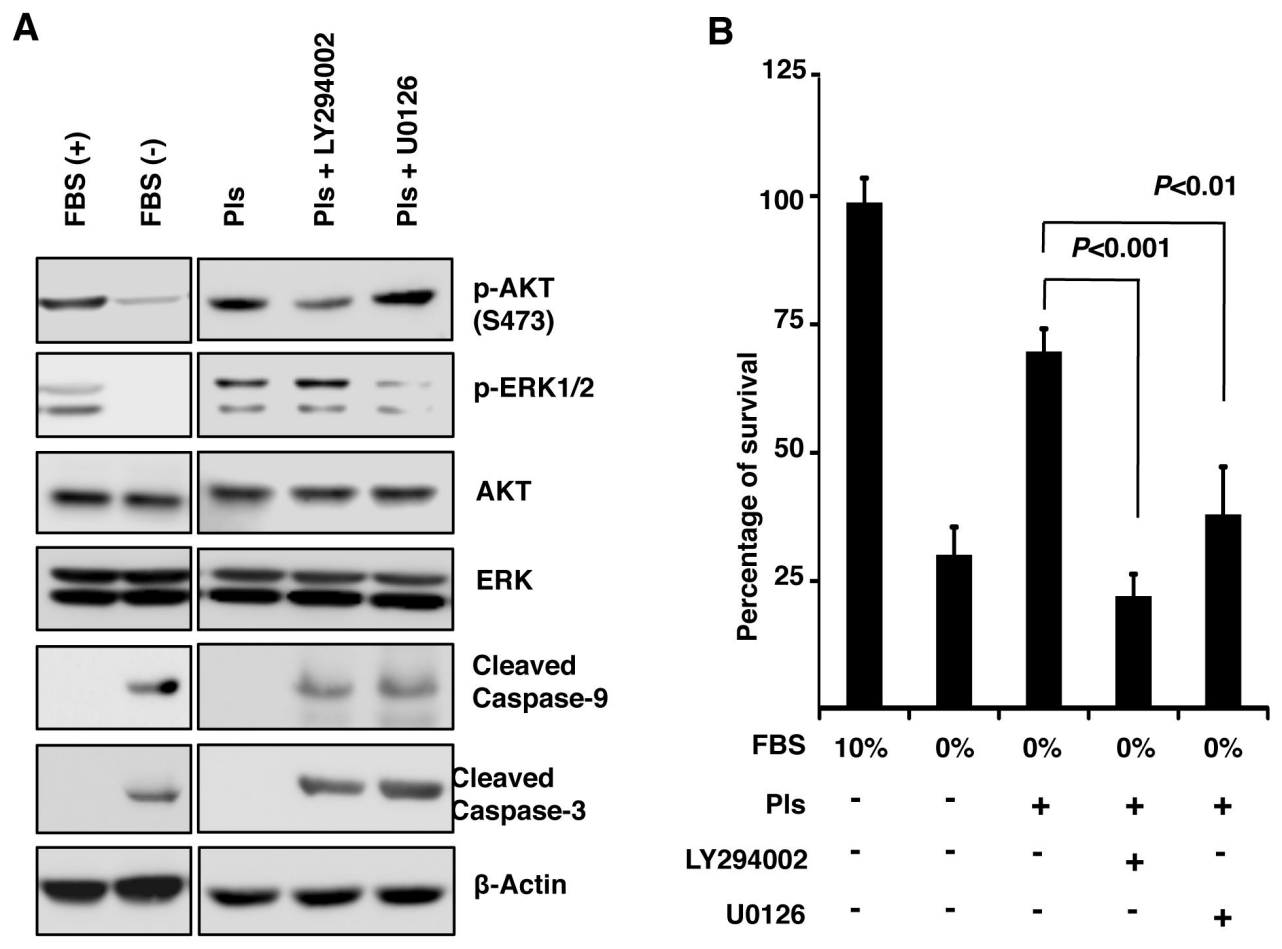
Plasmalogens-mediated cell survival was cancelled by PI3K and MEK kinases inhibitors. **A**) Specific inhibition of AKT and ERK1/2 phosphorylation by the inhibitors. Serum starved Neuro-2A cells were treated with Pls (5 µg/ml), Pls and LY294002 (5 µM) or Pls and U0126 (5 µM) for 24 hours. Western blotting data showed that the PI3K/AKT inhibitor LY294002 inhibited p-AKT (S-473) but not p-ERK1/2, while MEK inhibitor U0126 inhibited p-ERK1/2 but not p-AKT. Both inhibitors induced cleavage of caspase-9 and caspase-3. β-Actin was checked as loading control. The data represents more than three independent experiments. **B**) WST-8 assays. Neuro-2A cells were passaged in 96 well dishes (2000 cells/well) with 10% and 0% FBS containing MEM medium. Pls (10 µg/ml) were added to serum free groups and 12 hours later phosphoinositide 3-kinases (PI3Ks)/AKT inhibitor (LY294002) (5 µM), and MEK inhibitor (U0126) (5 µM) were added to the cells. After 24 hours of inhibitors treatments WST-8 assays were carried out to quantify survival cells. Each group, n=4, *P*<0.01 and 0.001, Bonferroni’s test).

Activation of AKT and ERK1/2 has been associated with inhibition of the apoptotic cleavages of caspase-9, caspase-3, and PARP-1 [15-17]. Protein tyrosine kinase receptors and G-protein coupled receptors are functionally associated with membrane lipid rafts. Pls are believed to present in the lipid rafts. We therefore hypothesized that Pls can modulate the signaling of AKT and ERK1/2. To clarify this, we cultured Neuro-2A cells in serum free medium for 12 hours and treated with Pls or vehicle for additional 12 hours. Interestingly, our experimental data showed that starvation-mediated decrease in phospho-AKT (p-AKT) and phospho-ERK1/2 (p-ERK1/2) was restored by Pls treatment (Figure 4A). Furthermore, quantification of the data in panel A showed a significant increase in p-AKT/AKT and p-ERK/ERK ratios following Pls treatment (Figure 4B, each group, n=4, *P*<0.001). In addition, time course experiments were carried out to identify the early time points of AKT and ERK activation by Pls administration. Following the Pls addition in the serum starved Neuro-2A cells, phosphorylation of AKT and ERK1/2 was enhanced in the earliest time points of 5 minutes (Figure 4C). Quantification of the specific signals in the panel C showed a significant gradual increase of AKT and ERK1/2 phosphorylation in the increasing time points (Figure 4D) (n=4, *P*<0.001). These evidences indicated that Pls might inhibit serum starvation-mediated apoptosis through the activation of AKT and ERK1/2. To confirm this, we have employed LY294002, which is known to inhibit AKT phosphorylation, and U0126, a MEK1/2 inhibitor. The doses of LY294002 and U0126 (5 µM each) used in the Pls treated cells were found to inhibit AKT and ERK1/2, respectively in a specific manner (Figure 5A). Cell proliferation studies (WST-8 assays) showed that the Pls-mediated cell survival was cancelled upon the inhibition of AKT and ERK1/2 (Figure 5B, each group, n=4, *P*<0.01 and 0.001). These evidences suggest that Pls enhance phosphorylation of AKT and ERK1/2, which can play an anti-apoptotic role in the neuronal cell death upon serum starvation. 

### Plasmalogens inhibit hippocampal neuronal cell death

**Figure 6:**
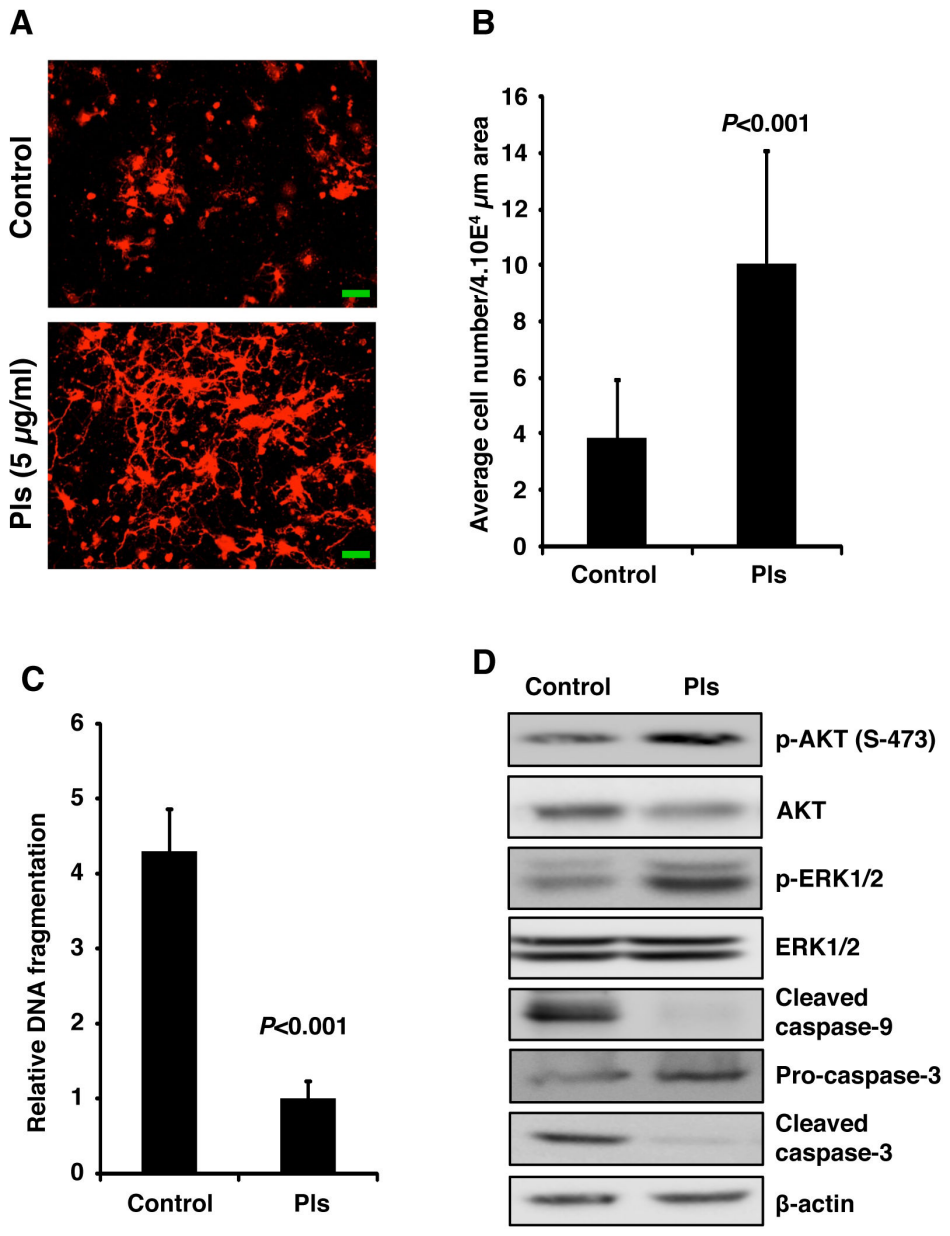
Plasmalogens inhibit hippocampal neuronal cell death *in* *vitro*. **A**) Pls treatments increase the survivability of primary hippocampal neurons. On DIV 3, hippocampal neurons were cultured with nutrient free medium and treated with vehicle (control) and Pls (5 µg/ml) for 72 hours. On DIV6, neuronal cells were stained by Dil (red color) and imaged by the fluorescence lifetime imaging microscope. Scale bar, 50 µm. **B**) The bars show the number of primary neurons in the specific area of randomly selected 12 different locations from each dish. The plotted data (average number ± SD) represents four independent experiments. Bonferroni’s test showed a significant difference between the two groups (each group, n=4, *P*<0.001). **C**) DNA fragmentation assays showed a significant reduction of DNA smearing in the Pls group compared with the vehicle control group (each group, n=4, *P*<0.001, Bonferroni’s test). **D**) Neuronal cell lysates (50 µg protein) of DIV6 were analyzed by western blotting. Pls treatments showed a reduction in the cleaved caspase-9 and caspase-3. Increased phosphorylation of AKT and ERK was also observed in the Pls treated primary neurons. These data represents three independent experiments.

To assay whether the anti-apoptotic effect of Pls can be observed in primary neurons, we have employed primary culture of mice embryonic (E18) hippocampal neurons. We have observed that withdrawal of B27 supplement on DIV 3 can reduce the number of survival neurons to about 50% on DIV 6 (data not shown). We then treated the neurons with Pls at the concentration of 5 µg/ml on DIV 3 and counted the survival neurons on DIV 6. A significant increase in the number of survival neurons was observed in the Pls treated neurons compared with that of nutrient deprived control neurons (Figure 6A and B, each group, n=4, *P*<0.001). To further confirm the apoptotic event we have analyzed the fragmentation assays and found a significant reduction of DNA fragmentation in the Pls group compared with that of the control group (Figure 6C, each group, n=4, *P*<0.001). Western blotting was also employed to investigate the changes in the cellular signaling and apoptosis pathways. Similar to Neuro-2A cells, Pls also enhance phosphorylation of AKT and ERK (Figure 6D). In our experimental condition, we did not find any change in total protein level of AKT and ERK, suggesting that Pls treatment could enhance phosphorylation status of these signaling proteins without affecting their total protein contents. Consistent with Neuro-2A cells, Pls treatment inhibited the cellular content of cleaved caspase-9 and caspase-3 in the primary neurons. Reduction of the uncleaved procaspase-3 in the control cells compared with that of Pls group further indicate that Pls treatment reduce the cleavage of procaspase-3 (Figure 6D). 

**Figure 7:**
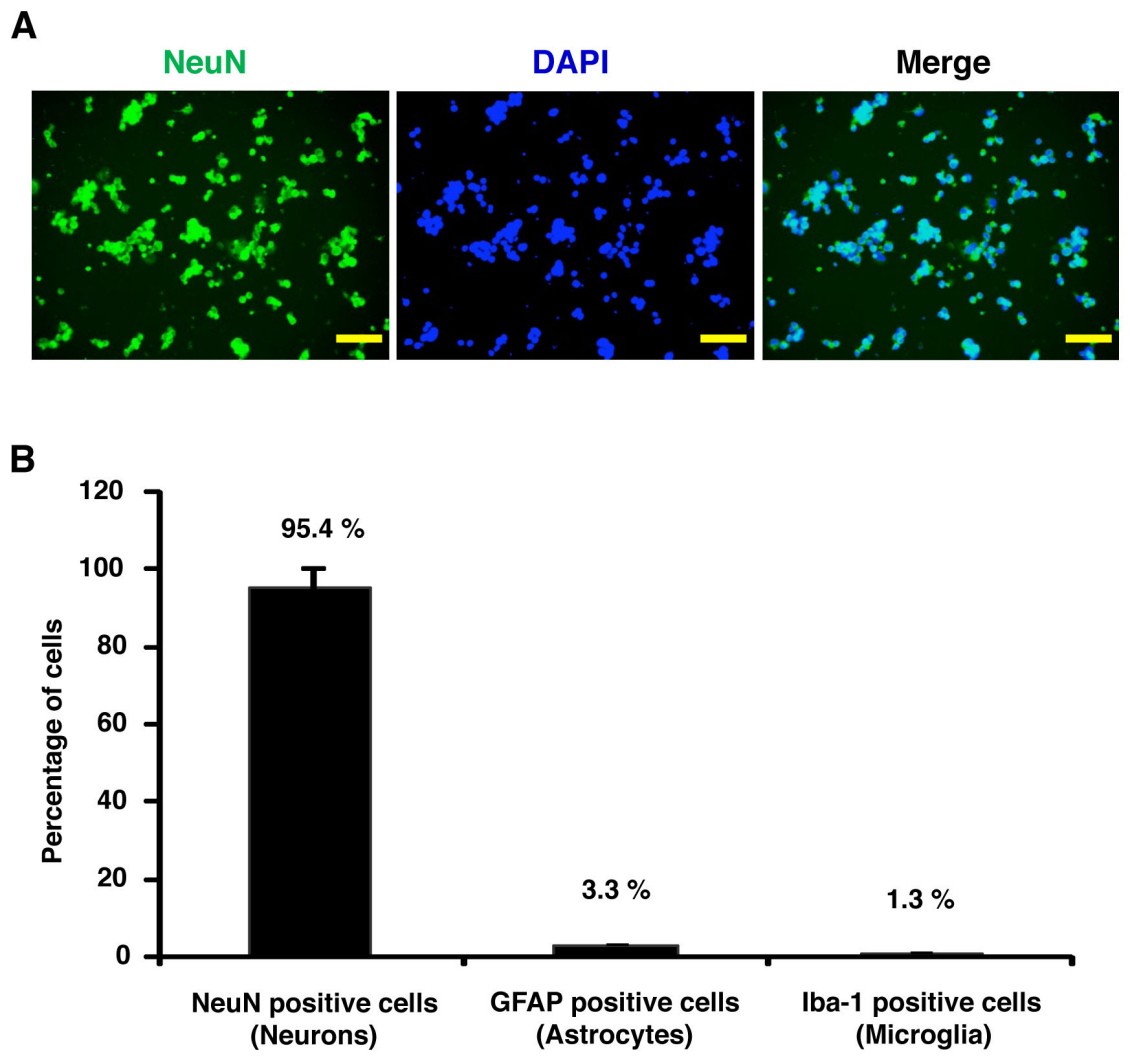
Primary hippocampal neurons were stained by the neuronal marker NeuN. **A**) To confirm the purity of *in* *vitro* primary hippocampal neuron culture, cells of DIV6 were stained by NeuN. DAPI was used to stain the cellular nucleus. Almost all the cells of DIV6 were stained by NeuN. Scale bar, 50 µm**. B**) Quantification of the DIV6 primary cultures cells stained by NeuN (neuron), GFAP (astrocytes), and Iba-1 (microglia). The data represents four independent experiments.

Primary neuron culture can often be contaminated by glial cells population. To avoid this contamination we have added Ara-C in the DIV3 neuronal culture. Ara-C is well known to inhibit glial proliferation in the primary culture. Furthermore, we have also stained the primary culture cells of DIV6 by markers for neurons (NeuN), astrocytes (GFAP) and microglia (Iba-1) and confirmed that more than 95 % of cells were neurons (Figure 7A and 7B).

### Plasmalogens do not prevent apoptosis of astrocyte-derived cells

**Figure 8:**
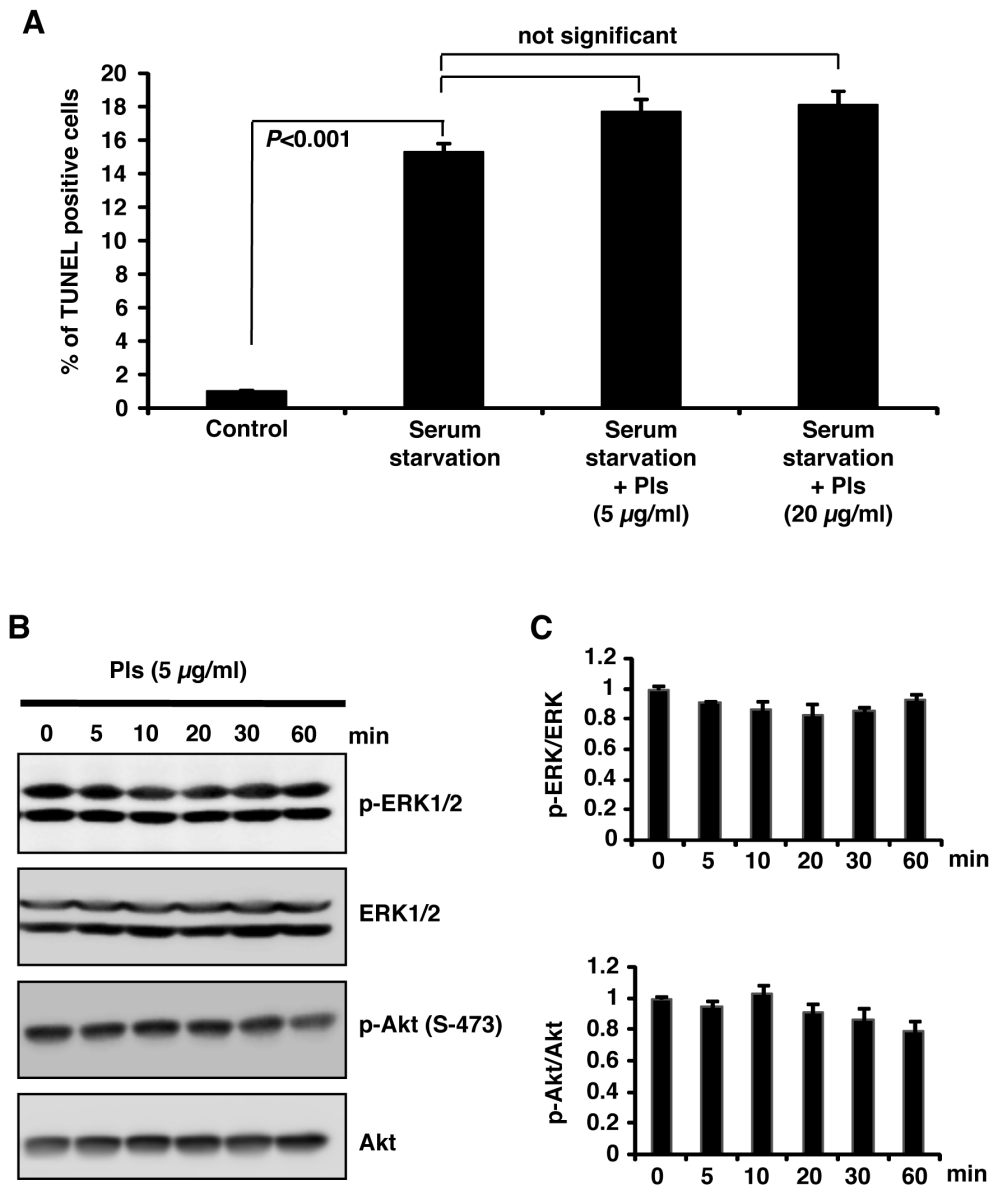
Pls cannot prevent apoptosis of astrocyte-derived cells and cannot increase phosphorylation of AKT and ERK. **A**) TUNEL assays showed no change in the number of apoptotic cells by Pls treatments. Astrocyte-derived A1 cells were cultured for 72 hours without serum in presence or absence of the different doses of Pls (5 and 20 µg/ml). The cells of the control group were cultured with 10% serum. **B**) Western blotting data showed no change in the phosphorylation of AKT and ERK by Pls (5 µg/ml) treatments. A1 cells were subjected to serum starvation for 12 hours followed by treatments with Pls in the indicated time points. **C**) Quantifications of the signals of panel B showed no change of phosphorylated AKT and ERK compared to their total protein levels.

To examine whether this anti-apoptotic effect of Pls is observed in other cells than neurons, astrocyte-derived A1 cells were cultured for 72 hours without serum in presence or absence of the different doses of Pls (5 and 20 µg/ml). As shown in Figure 8A, TUNEL assays showed no significant changes in the number of apoptotic cells by Pls treatments. Western blotting data showed no change in the phosphorylation of AKT and ERK by Pls (5 µg/ml) treatments during 60 min after serum starvation for 12 hours (Figure 8B). Quantifications of the signals of panel B showed no change of phosphorylated AKT and ERK compared to their total protein levels at any time points (Figure 8C). We therefore concluded that in our *in vitro* primary culture experiments, the effect of Pls was specific to neuronal cells.

### Plasmalogens inhibit Retinoic acid induced cell death

**Figure 9:**
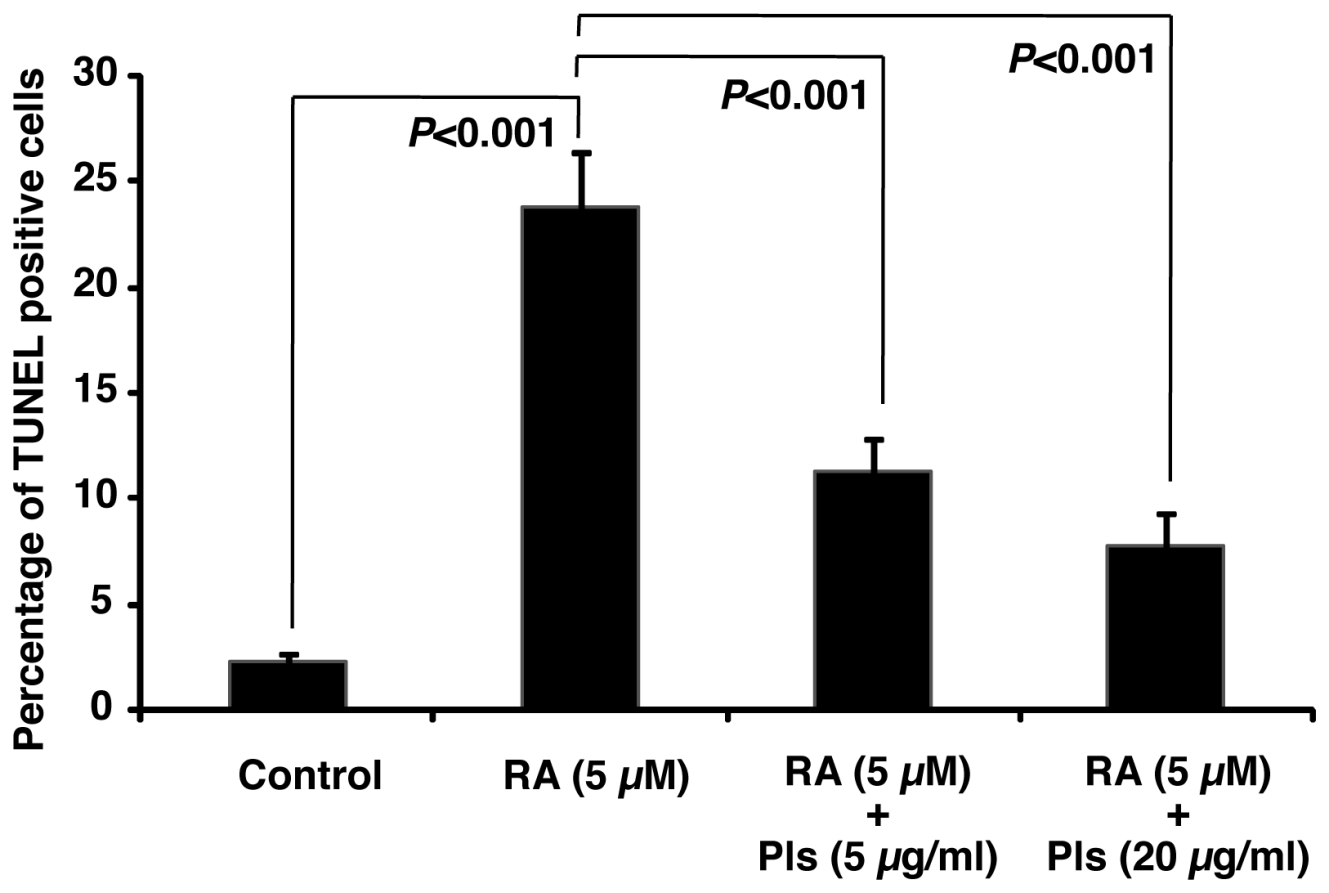
Retinoic acid-induced apoptosis of Neuro-2A cells was inhibited by Plasmalogens. Neuro-2A cells were cultured with 5 µM of Retinoic acid (RA) with or without Pls (5 and 20 µg/ml) for 72 hours. TUNEL assays were employed to quantify the apoptotic cells. A significant apoptosis inhibitory effect of Pls was obtained by both the concentrations of 5 and 20 µg/ml (n=4, *P*<0.001, Bonferroni’s test).

Retinoic acid (RA) is well known to inhibit cancer cell growth and can induce differentiation of neuroblastoma cells [18,19]. It is also known that RA can induce apoptosis in cancer cell lines [20,21]. To establish an apoptotic model we have treated the Neuro-2A cells with RA for 72 hours. A significant increase of apoptotic cells by the RA treatment was confirmed by TUNEL staining (Figure 9). Interestingly, Pls treatments significantly inhibited the apoptosis induced by RA (Figure 9). These evidences suggest that Pls not only inhibit the apoptosis mediated by serum starvation but it can also inhibit other apoptotic stimuli. 

## Discussion

Pls are the ether phospholipids that are found to be associated with neurodegenerative disorders and its precise biological role in the neurons is still elusive. Reduction of ethanolamine Pls in the brain, serum and erythrocyte membrane of AD patients was well reputed to be associated with the pathogenesis of AD [22-28]. Ceramide, which is a proapoptotic phospholipid, can reduce Pls by enhancing phospholypase A_2_ (PLA_2_) activity [29,30]. Therefore, we hypothesized that Pls have an anti-apoptotic activity. To our knowledge, the direct role of Pls in inhibiting neuronal cell death was unknown. We report for the first time that Pls can prevent neuronal cell death via a mechanism dependent on activation of AKT and ERK1/2. 

In AD pathogenesis, it was hypothesized that Pls can reduce membrane cholesterol level of the lipid rafts followed by the inhibition of γ-secretase activity resulting in the reduction of amyloid precursor protein (APP) processing [31]. We have also reported that Pls effectively inhibited microglial activation as well as accumulation of Aβ protein in the adult mice brains (3). These evidences suggest that Pls might work on cellular membrane to modulate membrane proteins function. In the present study, we have found that Pls enhance activation of AKT and ERK in neuronal cell lines. We still do not know the mechanism of how the Pls activate AKT and ERK. Therefore, further experiments will be carried out to address this issue in future. It is well known that activation of AKT and ERK occurs by the extracellular stimulation. Extracellular factors bind with the tyrosine kinase receptors present in the cell membrane and activate the downstream kinases leading to the phosphorylation of AKT and ERK [32]. Also many G-protein coupled receptors bind with the membrane to get functional and activate several kinases leading to the activation of AKT and ERK [32-34]. It is believed that lipid rafts present in the cellular membrane can play a major role in the activation of receptor tyrosine kinases and G-protein coupled receptors leading to the phosphorylation of AKT and ERK [32,35]. It is therefore possible that Pls can enhance activation of some tyrosine kinase receptors as well as G-protein coupled receptors by modulating their lipid raft distribution.

AKT and ERK are well known to inhibit apoptosis by inhibiting the activation of caspases. Several caspases were reported to be associated with neuronal cell death. Caspase-12 can enhance endoplasmic reticulum (ER)-mediated apoptosis and Aβ induced neurotoxicity [36,37]. Serum starvation increased caspase-12 protein level in mouse embryonic fibroblast cells [38]. However, caspase-12 was unaffected by serum starvation in Neuro-2A cells. It has been reported that, caspase-8 activation triggers neuronal cell death and serum starvation activates caspase-8 in adipocytes [39,40]. However, in our serum starvation model, caspase-8 was unchanged (Figure 3A). Caspase-9 is related to intrinsic apoptosis pathway and it has also been reported to be associated with neuronal cell death [41]. Interestingly, in our experiments the cleaved caspase-9 was increased significantly upon serum starvation, which was effectively suppressed by the addition of Pls (Figure 3). These evidences suggest that Pls can inhibit intrinsic apoptotic pathway to cancel serum starvation-mediated neuronal cell death. 

The precise signaling pathway of caspase-9 inhibition by Pls-induced phosphorylation of AKT and ERK are still elusive. Further studies will be conducted to address this in future. It has been reported that Cytochrome-C (Cyt-C), released from the mitochondria upon apoptotic stimuli, can activate caspase-9 [42]. The pro-apoptotic Bcl-2 family protein Bad can trigger the release of Cyt-C from the mitochondria that can result in activation of Casapse-9. AKT and ERK can phosphorylate the Bad protein to inactivate its proapoptotic function [43,44]. It is therefore, possible that Pls-mediated activation of AKT and ERK can inhibit caspase-9 probably by targeting Bad protein. It is to be noted that Cyt-C can also be released Bad independent pathway to activate caspase-9 [45]. In addition, caspase-9 can be cleaved independent of Cyt-C [20]. Therefore, future studies based on the common information could lead us to identify the precise mechanism of caspase-9 activation by Pls.

Interestingly, Pls treatments failed to inhibit apoptosis of astrocyte-derived cells and also there were no induction of AKT and ERK phosphorylation in the same cells (Figure 8). These important findings indicate that Pls-mediated activation of AKT and ERK could be prerequisite for anti-apoptotic function. Further study will be carried out to explore the detail mechanism of cell specific function of Pls. 

In conclusion, our cumulative evidences suggest that Pls activate AKT and ERK which are associated with the inhibition of neuronal cell death. This finding could lead us to make a hypothesis that the reduction of Pls in the brain of AD patients can be one of the reasons for neuronal loss. Future studies aimed to prove this hypothesis might help us to explore novel therapeutics to cure neurodegenerative diseases by preventing neuronal cell death.
